# Adverse reactions of piperacillin: A literature review of case reports

**DOI:** 10.1515/med-2024-0931

**Published:** 2024-04-10

**Authors:** Hongru Zhang, Liping Yang

**Affiliations:** Department of Pharmacy, ZhangJiakou First Hospital, Zhangjiakou City, Hebei Province 075000, China; Department of Pharmacy, Beijing Hospital, Beijing, China; National Center of Gerontology, Beijing, China; Institute of Geriatric Medicine, Chinese Academy of Medical Sciences, Beijing, China; Beijing Key Laboratory of Assessment of Clinical Drugs Risk and Individual Application (Beijing Hospital), Beijing 100730, China

**Keywords:** piperacillin, adverse drug reactions, case reports, toxic epidermal necrolysis, anaphylactic shock

## Abstract

**Aim:**

This study aimed to summarize case reports of adverse drug reactions (ADRs) caused by piperacillin and explore their effects on human organs in real-world settings.

**Method:**

Case reports of piperacillin ADRs were collected by searching databases such as PubMed, Embase, Web of Science, CNKI, WanFang, and VIP from inception to December 2022.

**Results:**

A total of 170 patients were ultimately included. The results revealed that ADRs caused by piperacillin were primarily associated with the entire body, followed by the blood system, skin and soft tissues, and the nervous system. The most frequently reported cases included anaphylactic shock, drug fever, rash, and thrombocytopenia. The most severe ADRs were identified as anaphylactic shock and bullous epidermal necrolysis. Furthermore, a comparison was made between systemic adverse reactions caused by piperacillin as a single drug and two composite preparations of piperacillin/β-lactamase inhibitor. ADRs not mentioned in the instructions included convulsions or hallucinations and Kounis syndrome (KS).

**Conclusion:**

This review suggests that the most severe ADRs associated with piperacillin are toxic epidermal necrolysis and anaphylactic shock. Rare ADRs caused by piperacillin, such as myoclonic jerks, hallucinations, and KS, were identified. The most common symptom with domestic preparations of piperacillin/sulbactam and piperacillin sodium was dyspnea.

## 1 Introduction

Piperacillin sodium is a semi-synthetic broad-spectrum penicillin antibiotic known for its efficacy against common Gram-negative, some Gram-positive, and anaerobic bacteria. Due to its susceptibility to hydrolysis by β-lactamase enzymes, it is often combined in 8:1 or 4:1 formulations with tazobactam or sulbactam to enhance its bactericidal activity against enzyme-producing bacteria. Given its broad antibacterial spectrum and stability, piperacillin combination preparations are frequently chosen as first-line treatments for complex infections [1]. However, the increased clinical use of piperacillin sodium and tazobactam sodium has also led to a rise in reports of adverse drug reactions (ADRs) [2,3]. Existing safety data for piperacillin largely derive from well-designed studies and scattered case reports, with a notable absence of real-world safety data. This study retrospectively analyzes case reports of piperacillin-related ADRs since its introduction, aiming to provide genuine safety data from clinical practice.

## 2 Methods

### 2.1 Search strategies

Computer-based searches were conducted in six databases, including PubMed, Embase, Web of Science, Wang Fang Date, CNKI, and VIP, covering the database’s inception until December 31, 2022. The search terms, both in Chinese and English, included “piperacillin” and “case reports,” connected using logical operators “and” and “OR,” as well as combined with subject headings and free terms (Box 1). Case reports documenting piperacillin ADRs were gathered, and relevant review articles were manually examined for additional references.

**Box 1:**　Search strategies

**Table:** 

#1 piperacillin[Title]
#2 case reports[Publication Type]
#3 piperacillin[Abstract]
#4 1 case[Title]
#5 #1 AND #2
#6 #1 OR #3 AND #4

### 2.2 Inclusion and exclusion criteria

Inclusion criteria were as follows: case reports of ADRs caused by piperacillin in clinical practice, encompassing individual case reports and series of case reports from which individual case information could be extracted.

Exclusion criteria were as follows: repetitive published reports, animal experiments, clinical trials, and secondary analysis literature; ADRs caused by other drugs; and inability to locate the original or full text.

### 2.3 Data extraction and processing

**Figure 1:**
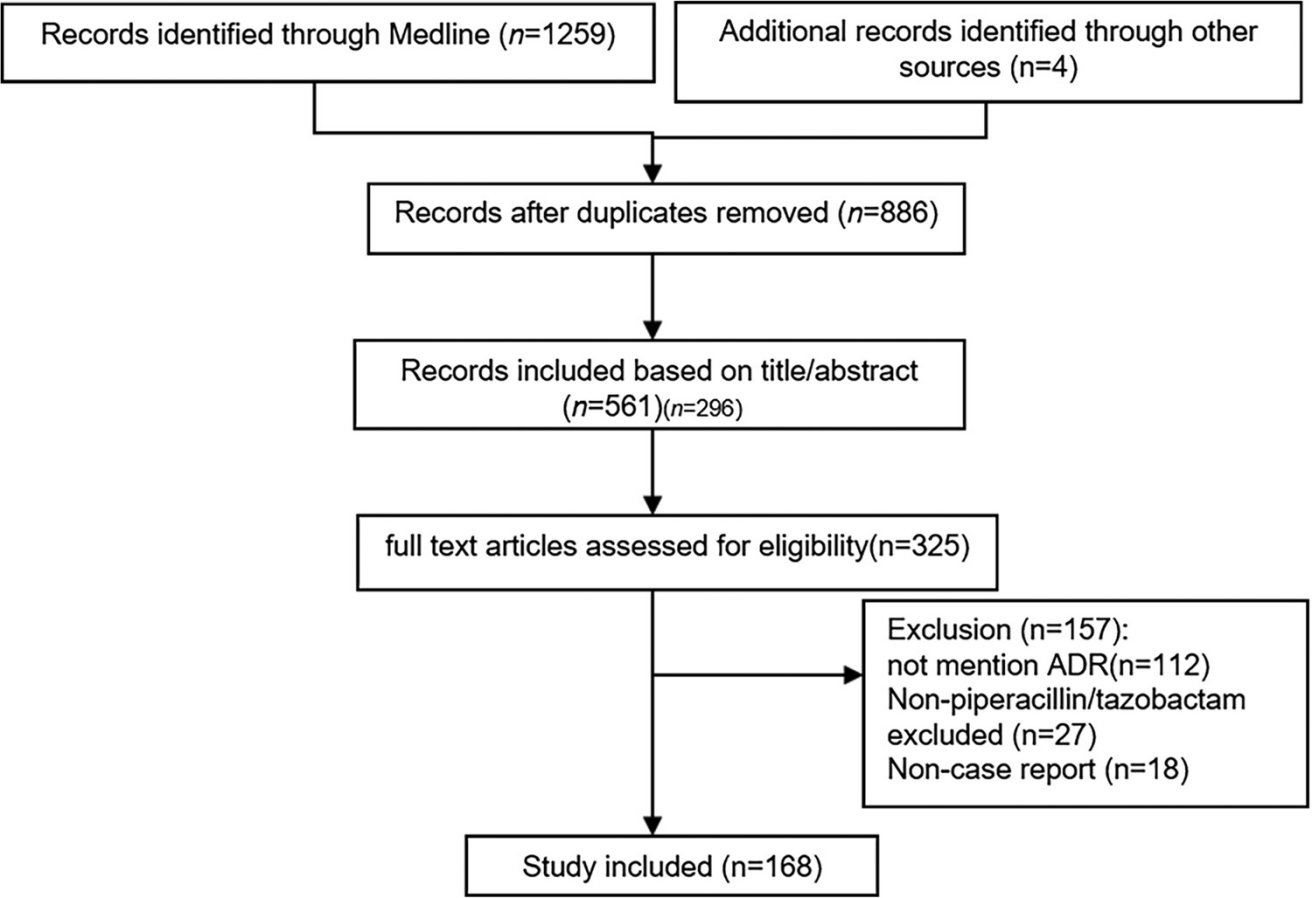
Flow chart illustrating the selection process of references.

The total number of cases was determined by summing the deduplicated data extracted from each database, as illustrated in Figure 1. Each case’s full text was carefully reviewed, and the extracted information included: (1) Basic information: year of publication, patient age, gender, race, underlying disease, all medications used, and their dosages as reported. (2) ADRs information: affected body parts, specific symptoms, onset time, duration, and time to resolution. (3) ADRs treatment: actions taken, treatment methods, reoccurrence, outcomes, and final status. The screening process was conducted independently by author Zhang, with multiple cross-checks to ensure data authenticity and reliability. Any disagreements were resolved through consultation with other researchers or relevant professionals. File organization and filtering were carried out using Endnote X6, while data extraction and analysis were performed using Excel.

## 3 Results

### 3.1 Basic information of cases

In this study, a total of 168 case reports were included (Figure 1), encompassing 170 patients, comprising 87 males and 83 females, resulting in a male-to-female ratio of 1.06:1. The median age was 54 years, with a range from 11 days to a maximum of 92 years. The patients were of Asian (*n* = 132) and Caucasian (*n* = 38) descent, as indicated in Table 1. The longest duration of piperacillin use was 30 days (*n* = 1), while the shortest duration was a single administration (*n* = 54), as shown in Table 2.

**Table 1:** Distribution of age and gender in patients

Age (years)	Male	Female	Total
	*n* (%)	*n* (%)	*n* (%)
0–11	8 (4.70)	9 (5.29)	17 (10.00)
12–17	3 (1.76)	1 (0.59)	4 (2.35)
18–44	17 (10.00)	22 (12.94)	39 (22.94)
45–64	30 (17.64)	25 (14.70)	55 (32.35)
65–80	22 (12.94)	17 (10.00)	39 (22.94)
≥80	7 (4.12)	8 (4.70)	15 (8.82)
Not recorded		1 (0.59)	1 (0.59)
Total	87 (51.18)	83 (48.82)	170 (100.00)

**Table 2:** Systems/organs and the clinical manifestations in 170 piperacillin ADRs

Duration of therapy	Num (*n*)	Ratio (%)	Main piperacillin-related ADRs in different systems/organs (*n*_1_)						
			Skin and soft tissue	Blood	Whole body	Nervous	Cardiovascular system	Gastro-intestinal	Others
<1 day	63	37.06	Rash (4), facial edema (4), angioneurotic edema (2)	—	Allergic shock (29), anaphylaxis (6)	Convulsion (7), ache of lower limb (4), rage (2)	Kounis syndrome (1), bradycardia (1), cardiac failure (1)	Abdominal pain (1)	Acute kidney injury (1)
1 day to 1 week	38	22.35	Rash (7), epidermal necrolysis (2), exfoliative dermatitis (2), Stevens-Johnson syndrome (1), Sweet syndrome (1), nail groove bleeding (1), angioedema angioedema (1), dress syndrome (1)	Thrombocytopenia (5), immune hemolytic anemia (2), decreased hemoglobin (1), eosinophilia (1), myelosuppression (1)	Drug fever (5), anaphylaxis (1)	Convulsion (2), hallucinate (1)	—	Diarrhea (2)	Renal damage (1)
1–2 weeks	40	23.53	Rash (5), Sweet syndrome (1), injection site necrosis (1)	Thrombocytopenia (6), leukocytopenia (4), immune hemolytic anemia (4), decreased hemoglobin (2), granulocytopenia (1), hemophagocytic lymphohistiocytosis (1), coagulation disordersn (1)	Drug fever (10)	Convulsion (1), hallucinate (1)	—	—	Hypokalemia (1), transaminase elevation (1)
2–4 weeks	26	15.29	Epidermal necrolysis (4), Rash (1), Dress syndrome (1)	Myelosuppression (5), thrombocytopenia (4), leukocytopenia (3), hemophagocytic lymphohistiocytosis (2), granulocytopenia (1), decreased hemoglobin (1)	Drug fever (4)	—	—	—	—
4–12 weeks	3	1.76	—	Myelosuppression (1), decreased hemoglobin (1), abnormal coagulation function (1)		—	—	—	—
>12 weeks	—	—	—	—	—	—	—	—	—

Among the 170 patients, 140 had clear documentation of piperacillin usage and dosage. Apart from one case involving bone cement filling, the rest received intravenous administration. Of the 139 patients receiving intravenous administration, piperacillin sodium and tazobactam sodium were used in 98 cases, piperacillin sodium and sulbactam sodium in 22 cases, and piperacillin sodium alone in 9 cases.

### 3.2 System or organ involved and main clinical manifestations of piperacillin ADRs

Among the 170 reported patients, ADRs caused by piperacillin-containing preparations predominantly affected the entire body (*n* = 55), the blood system (*n* = 48), the skin and soft tissue system (*n* = 39), the nervous system (*n* = 18), the gastrointestinal system (*n* = 3), the cardiovascular system (*n* = 3), the kidney and urinary system (*n* = 2), and the hepatobiliary system (*n* = 1). Additionally, there were instances of hypokalemia (*n* = 1). The affected systems/organs and primary clinical manifestations are detailed in Table 3.

**Table 3:** Distribution of administration time

Systems/organs	Num (*n*)	Ratio (%)	Mainly ADRs (*n*_1_)	The median time of duration (day)	The median time of ADRs occurrence (day)	The median time ADRs disappeared (day)
Whole body	55	32.35	Allergic shock (29)	(One time, two times)	(One time, two times)	(10 min, 2)
			Drug fever (19)	9.5(3, 19)	8.5(2, 19)	2(1, 7)
			Anaphylaxis (7)	(One time, 2)	(One time, 2)	(10 min, 3)
Blood	48	28.24	Thrombocytopenia (15)	9(1, 20)	8(1, 22)	4(2, 15)
			Leukocytopenia (7), granulocytopenia (2)	14(8, 21)	14(8, 19)	1(1, 8)
			Immune hemolytic anemia (6), decreased hemoglobin (5)	9(6, 30)	9(4, 30)	6(2, 14)
			Myelosuppression (7), hemophagocytic lymphohistiocytosis (3), coagulation disorders (2), pneumonia with eosinophilia (1)	17(2, 30)	14(2, 25)	4(2, 10)
Skin and soft tissue	39	15.88	Rash (17)	(One time, 15)	(One time, 13)	(5 min, 14)
			Epidermal necrolysis (6), dermatitis exfoliativa (2), dress syndrome (2), sweet syndrome (2), Stevens-Johnson syndrome (1),	6(2, 20)	4.5(1, 30)	7(4, 14)
			Facial edema (4), angioneurotic edema (3), nail groove bleeding (1), injection site necrosis (1)	(One time, 9)	(One time, 9)	(30 min, 10)
Nervous	18	8.82	Convulsion (10), ache of lower limb (4), hallucinate (2), restless (2)	(One time, 11)	(One time, 11)	(5 min, 5)
Others	10	4.70	Diarrhea (2), abdominal pain (1), renal damage (2), hypokalemia (1), transaminase elevation (1), Kounis syndrome (1), transaminase elevation (1), hypokalemia (1)	(One time, 12)	(One time, 12)	(45 min, 26)

The primary manifestations in the 55 patients with ADRs involving the entire body were allergic shock (*n* = 29), most of which occurred during the initial infusion (*n* = 28). The onset time of allergic shock was from 10 min after infusion to the second dose. The resolution time for ADRs varied from 10 min to 2 days. Tragically, two patients succumbed to severe anaphylactic shock despite rescue efforts.

In addition, drug fever developed in 19 patients after medication, with some cases occurring after white blood cell counts, CRP, and other infection indicators had returned to normal (*n* = 5). The median onset time was 8.5 days, ranging from 2 to 19 days, and the median duration of ADRs was 2 days, ranging from 1 to 7 days. With the exception of four cases treated symptomatically with dexamethasone or ibuprofen, the body temperature of the remaining patients quickly returned to normal after discontinuation of the drug.

Seven patients experienced various symptoms such as chills, general numbness, itching, and systemic allergic reactions after drug use, with six cases occurring after a single dose and one case occurring after 2 days of use. ADR symptoms ceased with symptomatic treatment, and the symptoms subsided within 10 min to 3 days.

Among the 48 patients with hematological ADRs, the predominant manifestation was thrombocytopenia (*n* = 15). The median onset time was 8 days, ranging from 1 to 22 days, and the median duration of ADRs was 4 days, ranging from 2 to 15 days. Leukopenia or neutropenia occurred in nine cases, with a median onset time of 14 days, ranging from 8 to 19 days, and a median duration of 1 day, ranging from 1 to 8 days. Immune hemolytic anemia was observed in six cases, and decreased hemoglobin in five cases, with a median onset time of 9 days, ranging from 4 to 30 days, and a median duration of ADRs of 6 days, ranging from 2 to 14 days. Myelosuppression (*n* = 7), hemophagocytic lymphohistiocytosis (*n* = 3), coagulopathy (*n* = 2), and eosinophilic pneumonia (*n* = 1) were also reported. These hematological symptoms generally improved following drug withdrawal and symptomatic treatment. Among patients with hematological ADRs, 24 had underlying renal dysfunction. Seven of those with decreased hemoglobin or immune hemolysis had underlying cystic fibrosis, and three patients with hemophagocytic lymphohistiocytosis were children under 10 years old.

Among the 39 patients with ADRs in the skin and soft tissue system, the primary manifestations included rash (*n* = 17). The onset time ranged from 1 to 13 days, and the duration of ADRs ranged from 5 min to 14 days. There were six cases of epidermal necrolysis, two cases of exfoliative dermatitis, two cases of Dress syndrome, two cases of Sweet syndrome, and one case of Stevens-Johnson syndrome. Facial edema (*n* = 4), angioneurotic edema (*n* = 3), nail groove hemorrhage, and injection site necrosis were each observed in one case. Three patients succumbed to severe epidermal necrolysis, with two of them being older than 65 years old and having underlying diseases, while one was less than 1 year old.

The 18 patients experiencing ADRs of the nervous system primarily exhibited symptoms such as convulsions (*n* = 10), lower limb pain (*n* = 4), hallucinations (*n* = 2), and irritability (*n* = 2). The onset of these symptoms ranged from 1 to 11 days, and the duration of ADRs varied from 5 min to 5 days. Notably, 11 of these patients were older than 65 years, constituting 61.11% of the total number of neurological ADRs. Two patients with hallucinations were both older than 65 years and had renal insufficiency. It is worth mentioning that convulsions and hallucinations are not listed in the drug instructions.

Other ADRs included diarrhea or abdominal pain (*n* = 3), renal injury (*n* = 2), bradycardia (*n* = 1), heart failure (*n* = 1), Kounis syndrome (KS) (*n* = 1, unreported), elevated transaminase (*n* = 1), and hypokalemia (*n* = 1). Notably, KS was not mentioned in the drug instructions.

### 3.3 Statistics and comparison of allergic shock and systemic allergic reaction of different preparations of piperacillin

We conducted a statistical comparison of symptoms and specific preparations among 36 patients who experienced allergic shock and systemic allergic reactions. This group included 14 cases involving piperacillin sodium and tazobactam sodium, 14 cases with piperacillin sodium and sulbactam sodium, and 8 cases with piperacillin sodium. The most common symptoms were dyspnea (*n* = 12), followed by disturbance of consciousness (*n* = 9), general itching (*n* = 8), cardiac arrest (*n* = 2), facial cyanosis (*n* = 2), chills and fever (*n* = 1), facial edema (*n* = 1), and decreased blood pressure (*n* = 1). Refer Table 4 for more details.

**Table 4:** Correlation between anaphylaxis and drug preparations

Dosage form	Trade name	Manufacturer	Usage and dosage	Cardinal symptom	The time of ADRs occurrence	Num (*n*)
Piperacillin sodium/tazobactam sodium (14)	Zosyn(8:1)	Wyeth Lederle S.R.L	4.5 g Tid	Generalized rash with pruritus, faver	2 days	1
		North China Pharmaceutical (4:1)	2.5 g	Chest distress, disturbance of consciousness	One time	1
			3.75 g	Palpitations and chest tightness, disturbance of consciousness	One time	1
	1.125 g(8:1)	Qilu Tianhe Pharmaceutical Co., Ltd	3.375 g	Nausea and vomiting, pruritus/dyspnea, facial edema	One time	2
		Qilu Pharmaceutical Co., Ltd	2.25 g	Unconsciousness, gatism	One time	1
	Zoppen(4:1)1.25 g	Zhejiang AngLiKang Pharmaceutical Co., Ltd	2.5 g	Unconsciousness, vomiting, cyanosis, increased eye and nose secretions	One time	1
	Tazocin(8:1)2.25 g	Zhuhai Federal Pharmaceutical Co.	4.5 g	The whole body was flushed with itching and blurred vision	One time	2
		Unspecified	4.5 g	Pallor, pruritus	One time	2
		Unspecified	4.5 g	Unconsciousness, cyanosis	One time	1
		Unspecified	Unknown	Pruritus without rash, sweating profusely, nausea, vomiting	One time	2
Piperacillin sodium/sulbactam sodium (14)	Li Ke Duo(4:1)	Sichuan Pharmaceutical	2.5 g	Tingling of the mouth, unconsciousness	One time	2
			0.75 g	Unconsciousness, pallor	One time	1
	Pai Shu(4:1)	Shanghai Shangyao Xinya Pharmaceutical Co., Ltd	3.75 g	Abdomen pain with diarrhea, cardiac arrest	One time	1
			1.25 g	Celialgia and vomiting, cardiac arrest	One time	1
	Xintemie(2:1)	Xiangbei Wei’erman Pharmaceutical Co., Ltd	1.5 g	Facial edema, cyanosis	One time	1
			3.0 g	Dyspnea, acute hypotension	One time	2
	Yi Tan(4:1)	Harbin Pharmaceutical Group	5.0 g	The extremities were cold, acute hypotension, elevation of cardiac enzymes	One time	1
			2.5 g	Unconsciousness, pallor	One time	1
	Bai Ding(4:1)	Shandong Ruiyang Pharmaceutical Co., Ltd	3.75 g	Dyspnea, headache and nausea, pallor	One time	1
		Unspecified	Unknown	Dyspnea, general numbness	One time	2
		Unspecified	3 g	Dyspnea, cyanosis	One time	1
Piperacillin sodium (8)	Tai Yue da Kang	Shanxi Zhendong Taisheng pharmaceutical Co., Ltd	Skin test	Dyspnea, cyanosis	One time	1
	Yi Mie	Shandong Bokang pharmaceutical Co., Ltd	6.0 g	Dyspnea, acrocyanosis	One time	2
			2.0 g	Tingling of the mouth with cyanosis, rash	One time	1
		Unspecified	7.0 g	Numbness in hands and feet, dyspnea, unconsciousness	Two times	1
		Unspecified	5.0 g	Facial edema with cyanosis, rash	One time	1
		Unspecified	4.0 g	Chest tightness, holding breath, dyspnea	One time	1
		Unspecified	2.0 g	Chills and fever	One time	1

Among the 14 patients treated with piperacillin sodium and tazobactam sodium, the primary symptoms were pruritus in 8 cases, disturbance of consciousness in 5 cases, and dyspnea in 1 case. In the 14 patients who used piperacillin sodium and sulbactam sodium, dyspnea was the most common symptom, occurring in 6 cases, followed by 4 cases of disturbance of consciousness, 2 cases of cardiac arrest, 1 case of facial edema, and 1 case of blood pressure drop. Among the eight patients treated with piperacillin sodium, five experienced dyspnea, two had facial cyanosis, and one had chills and fever.

Upon comparison, it is evident that systemic itching symptoms only appeared in patients using piperacillin sodium and tazobactam sodium. Dyspnea was the most common symptom with domestic preparations of piperacillin sulbactam, and there were no reported adverse reactions associated with piperacillin sodium.

### 3.4 Treatment of ADRs

Upon the occurrence of ADRs, 169 out of 170 patients discontinued the use of piperacillin-containing preparations. The remaining patient had received an implantation of piperacillin-containing bone cement and did not experience any additional adverse reactions after receiving symptomatic treatment, thus not requiring any specific intervention. Among the treatment approaches employed: 41 patients were solely discontinued from or switched to other antibiotics for infection control; 86 patients received intravenous steroid treatment, including medications such as methylprednisolone, dexamethasone, or hydrocortisone. Two patients underwent topical steroid therapy. Other major treatment measures included the administration of leukocyte-enhancing drugs (*n* = 10), intravenous immunoglobulin (IVIG) (*n* = 8), the use of antipyretic analgesic drugs like ibuprofen (*n* = 7), infusion of blood products such as plasma, hemoglobin, or platelets (*n* = 5), and hemodialysis (*n* = 4).

### 3.5 Death cases

Out of the 170 cases of ADRs induced by piperacillin-containing preparations, six cases resulted in fatalities. These included three cases of severe epidermolysis bullosa, two cases of ineffective rescue in the context of anaphylactic shock, and one case of severe pneumonia. It is important to note that the patient with severe pneumonia had metastatic renal cell carcinoma, and their death may not have been directly related to piperacillin [4]. This data underscores that epidermolysis bullosa and anaphylactic shock are the primary causes of fatalities associated with piperacillin preparations.

## 4 Discussion

### 4.1 Statement of key findings

The ADRs induced by piperacillin preparations in this study encompassed a wide range of organs and systems, including the whole body, blood system, skin and soft tissue system, nervous system, gastrointestinal system, liver, kidney, heart, and electrolyte balance. Systemic ADRs included allergic shock, allergic reactions, and drug fever, which could be swiftly alleviated after discontinuation of the drug and symptomatic treatment. Blood system ADRs involved reductions in various types of blood cells, with the most common being a decrease in platelet and white blood cell counts [3]. Skin and soft tissue ADRs were mainly characterized by rash or localized edema, generally of mild severity, and ameliorated after discontinuing the drug and administering local treatments. Neurological ADRs were diverse, including convulsions, irritability, lower limb pain, and hallucinations, and their occurrence may be associated with advanced age and underlying kidney diseases. Other less frequent ADRs encompassed gastrointestinal reactions, liver or renal dysfunction, bradycardia, heart failure, and hypokalemia. Notably, this study identified previously unreported ADRs such as piperacillin-induced KS, as well as convulsions and hallucinations that were not mentioned in the drug instructions. Furthermore, different piperacillin preparations exhibited variations in the types and frequencies of allergic reactions. Epidermal necrolysis was identified as a high-mortality ADR warranting significant attention.

### 4.2 Strengths and weaknesses

We have analyzed case reports of adverse reactions to piperacillin and identified both severe and non-specific reactions with low incidence rates. Epidermal necrolysis was found to have a high mortality rate, while KS and nervous system reactions like convulsions and hallucinations were not mentioned in the manual. Additionally, we compared the systemic adverse reactions of various piperacillin preparations and observed differences in adverse reactions among different compound preparations.

There are some limitations in this study: (1) our study includes only case reports from the database, which may lead to statistical bias as the collected ADRs may be more severe, rarer, or more common in the population. This limitation prevents us from reflecting the real-world situation accurately. (2) The small sample size and inevitable lack of information in case reports may affect the credibility of our conclusions. For instance, the analysis and comparison of the occurrence ratio of allergic reactions caused by different piperacillin preparations could be affected. (3) Despite piperacillin preparations being on the market for many years, we found a limited number of case reports from English databases, which affects the scientific reliability of our statistical results.

### 4.3 Interpretation

Piperacillin, a semi-synthetic urea penicillin antibiotic used in the treatment of infectious diseases, can lead to ADRs involving multiple systems or vital organs [5,6]. Notably, the instructions do not mention KS associated with piperacillin and its enzyme compound preparations. KS is characterized by acute coronary syndrome triggered by mast cell and platelet activation during anaphylaxis or allergic reactions. Before 1991, Kounis and Zavras referred to it as “allergic angina” and “allergic myocardial infarction” [7,8]. KS can be induced by various factors, including hymenoptera insect bites, food allergies, and drugs. Some drugs reported to cause KS include contrast agents, intravenous anesthetics, and non-steroidal anti-inflammatory drugs [7,8]. KS is a severe medical emergency often underestimated in clinical practice [9]. While it shares symptoms like chest pain and tachycardia with allergic shock, the treatment approaches differ significantly. Allergic shock is typically treated with adrenaline and dexamethasone, whereas KS treatment commonly involves intravenous steroids (76%), nitroglycerin (47%), H1-blockers (70%), and H2-blockers (35%) [10]. The use of epinephrine in KS treatment remains controversial due to its potential to worsen cardiac ischemia, prolong QT characteristics, induce arrhythmia, and, importantly, promote atherosclerotic plaque rupture and sudden blood pressure increases, exacerbating the condition. Nitrates may offer greater benefits to KS patients [9,11,12]. Despite KS not being a rare adverse reaction, timely and accurate diagnosis, along with correct treatment, are essential to mitigate serious consequences. Medical professionals must enhance their understanding of this condition.

In this study, a significant proportion of patients experienced convulsions or hallucinations, accounting for two-thirds of all patients with ADRs in the nervous system. It is noteworthy that these patients were predominantly elderly and often had underlying kidney diseases. Numerous studies have explored the impact of piperacillin and other penicillin antibiotics on the nervous system. The underlying mechanism is the structural similarity between the β-lactam ring in piperacillin and the inhibitory neurotransmitter γ-aminobutyric acid. Factors contributing to piperacillin-related neurotoxicity include excessive usage, impaired renal function, advanced age, concurrent central nervous system conditions, and the simultaneous use of nephrotoxic drugs [13–15]. Fortunately, such adverse reactions can be rapidly alleviated upon discontinuation of piperacillin, typically without causing severe consequences. However, caution should be exercised when prescribing piperacillin to patients with central nervous system diseases, as it may exacerbate their condition. Additionally, our study observed that patients experiencing convulsions or hallucinations were all treated with piperacillin in combination with enzyme compound preparations. Interestingly, there were no reports of altered consciousness resulting from piperacillin sodium alone in systemic ADR case reports. This suggests that β-lactamase inhibitors may enhance the effect of piperacillin on the nervous system, although the specific mechanism remains unreported.

Allergic reactions triggered by piperacillin preparations are primarily attributed to impurities generated during piperacillin degradation. Piperacillin’s fundamental structure is 6-aminopenicillanic acid, which has poor stability and readily degrades into substances like penicilloic acid, penicillothiazolin, and 6-aminopenicillanic acid polymers. These degradation products can act as antigens or haptens, causing allergic reactions [6,16,17]. Importantly, our study found that domestic piperacillin sodium and tazobactam sodium had higher impurity levels compared to imported preparations, resulting in varying incidences and severity of allergic reactions after administration [18]. Among the allergic reactions reported in our study, only one case was associated with the use of imported piperacillin sodium and tazobactam sodium, presenting symptoms of systemic rash with high fever. In contrast, the remaining allergic reactions were linked to domestic piperacillin preparations and exhibited more severe symptoms. This disparity may be linked to the drug preparation process, aligning with the findings of Tian Li’s research [18].

Epidermal necrolysis is a severe drug-induced skin and mucosa reaction characterized by blisters and widespread epidermolysis. It can also involve multiple organ systems such as the liver and kidneys. During the acute phase, it may lead to multiple organ failure, resulting in a high mortality rate. Notably, individuals over the age of 40 and those with epidermal lysis affecting more than 10% of their body surface area are at a higher risk of death [19]. The “Expert consensus on diagnosis and treatment of Stevens-Johnson syndrome/toxic epidermal necrolysis” recommends treatment options that include wound management, nutritional support, and medication with glucocorticoids, IVIG, cyclosporine, and TNF-α antagonists [20]. In our study, we summarized six patients with epidermal necrolysis, and unfortunately, three of them succumbed to the condition. Despite three patients receiving good nutritional support and drug treatment, they still experienced multiple organ failure due to the rapid progression of the disease [21–23]. This underscores the critical nature of epidermal necrolysis, with a very high mortality rate once symptoms occur. Therefore, it is essential to remain vigilant for such ADRs when using piperacillin to prevent serious consequences.

### 4.4 Further research

Comprehensive data will be pursued by including various types of reports on piperacillin adverse reactions in the later stages, aiming to enhance the reliability of statistical incidence data. Furthermore, the effects of adverse reactions and their mechanisms caused by different enzyme inhibitors will be explored.

## 5 Conclusion

In summary, serious and fatal ADRs associated with piperacillin-containing preparations primarily include allergic shock and epidermal necrolysis. The most common ADRs involve the entire body and blood system. Notably, KS has only been reported in a single case, while convulsions and hallucinations, as ADRs, are not mentioned in the instructions for piperacillin preparations. ADRs related to piperacillin in the nervous system are more prevalent among patients with impaired renal function and advanced age, with an increased risk associated with the use of enzyme inhibitors. Epidermal necrolysis represents a particularly severe ADR with a high mortality rate.
